# Z-disc Transcriptional Coupling, Sarcomeroptosis and Mechanopoptosis

**DOI:** 10.1007/s12013-012-9430-6

**Published:** 2012-10-14

**Authors:** Ralph Knöll, Byambajav Buyandelger

**Affiliations:** Myocardial Genetics, British Heart Foundation—Centre of Research Excellence, National Heart & Lung Institute, Imperial College, Hammersmith Campus, London, UK

**Keywords:** Cell death, Apoptosis, Myocardial function, Adaptation

## Abstract

Cardiovascular diseases are the leading cause of morbidity and mortality worldwide. Heart failure, which contributes significantly to the incidence and prevalence of cardiovascular-related diseases, can be the result of a myriad of diverse aetiologies including viral infections, coronary heart disease and genetic abnormalities—just to name a few. Interestingly, almost every type of heart failure is characterized by the loss of cardiac myocytes, either via necrosis, apoptosis or autophagy. While the former for a long time mainly has been characterized by passive loss of cells and only the latter two have been regarded as active processes, a new view is now emerging, whereby all three forms of cell death are regarded as different types of programmed cell death which can be induced via different stimuli and pathways, most of which are probably not well understood (Kung et al., Circulation Research 108(8):1017–1036, 2011). Here, we focus on the sarcomeric Z-disc, Z-disc transcriptional coupling and its role in pro-survival pathways as well as in striated muscle specific forms of cell death (sarcomeroptosis) and mechanically induced apoptosis or mechanoptosis.

## Introduction

**Fig. 1:**
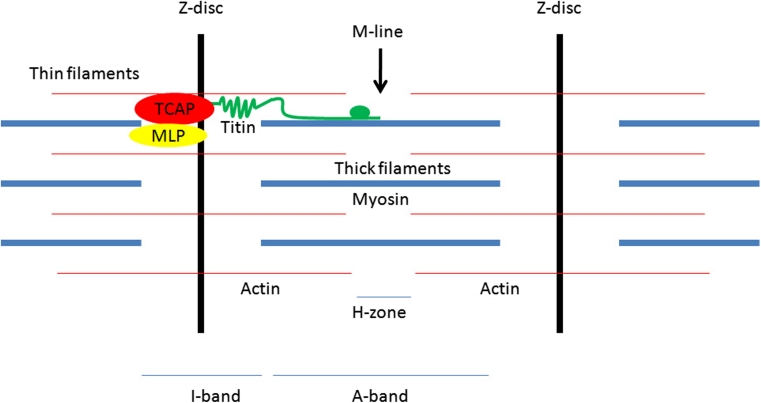
Schematic diagram of a sarcomere

The heart is a dynamic organ capable of self-adaptation to various changes in mechanical demands, but the underlying molecular mechanisms remain not well understood. Sarcomeres, the smallest functional units in striated muscle, are laterally demarked by Z-discs—structures composed of probably hundreds of different proteins and which represent one of the most complex macromolecular structures found in biology. Z-discs undergo conformational changes on a constant basis: from the basket weave structure during systole to the small square lattice during diastole, but the biological functions assigned to these structural changes remain largely elusive (Fig. 1).

During the last decades, a multitude of novel Z-disc proteins and their interacting partners have been identified, which has led to the identification of new and unsuspected functions and which have now been assigned to this structure. This includes the importance of Z-discs for intracellular signalling, including phosphorylation and dephosphorylation as well as acetylation and deacetylation or other posttranslational modifications. Z-discs are involved in mechanosensation and mechanotransduction, they are important for protein turnover including autophagy, and they are linked to the t-tubular system as well as to the sarcoplasmic reticulum and therefore to calcium metabolism. Moreover, the discovery of various mutations in a number of Z-disc proteins, which lead to perturbations of several of the above-mentioned systems, give rise to a diverse group of diseases which have been termed Z-discopathies and which include a wide range of cardiomyopathies and muscular dystrophies (please see for a recent review [1]).

However, our understanding of the precise molecular mechanisms which link Z-disc-related molecular events to short- and long-term effects remains incomplete. Here, we mainly focus on telethonin and other Z-disc proteins and how these proteins may be linked to muscle function and pro-survival pathways to avert apoptotic types of cell death.

## Telethonin and p53

Telethonin (TCAP) is a 167 amino-acid, striated muscle-specific expressed Z-disc protein with a unique β-sheet structure and no direct homologue genes. It binds in an antiparallel (2:1) sandwich complex to the titin Z1–Z2 domains, linking together the N-termini of two adjacent titin molecules [2]. This interaction also represents the strongest protein–protein interaction observed to date [3]. Beside its interaction with titin, telethonin interacts with a wide variety of different proteins. In this context, telethonin is phosphorylated by protein kinase D [4] and is also known as an in vitro substrate of the titin kinase, an interaction thought to be critical during myofibril growth [5]. The giant elastic protein titin extends across half the length of a sarcomere and is thought to stabilize sarcomere assembly by serving as a scaffold to which other contractile, regulatory and structural proteins attach [6]—therefore, by interacting with two different titin domains, telethonin may well affect several titin mediated processes.

Also, telethonin was shown to physically partner with muscle LIM protein (MLP, CSRP3), hypothesized to be part of a macromolecular mechanosensor complex and to play a role in a subset of human cardiomyopathies [7–9]. In addition, telethonin interacts with calsarcin-1 (also known as FATZ-2 or myozenin-2), a cardiomyopathy candidate gene [10] known to affect hypertrophic signalling via modulation of calcineurin activity [11]. Telethonin also may be linked to hypertrophic signalling via its interaction to myostatin (also known as GDF8) [12], a negative regulator of cell growth, and via its interaction with bone morphogenetic protein 10 (BMP10), a protein able to promote growth of cardiac myocytes [13]. Another important finding is that telethonin expression is inducible by stretch force and that integrin-linked kinase (ILK), also important in mechanosensory processes [9], negatively regulates telethonin expression [14].

Moreover, telethonin may well play an important role in various types of cancer. In this context, telethonin has been reported to interact with MOST1 (C8orf17), a protein ubiquitously expressed in many cancer cell lines [15].

Also, telethonin interacts with ankyrin repeat protein 2, which may provide a link to titin’s I-band-mediated signalling. Telethonin’s interaction with small ankyrin-1 (a transmembrane protein of the sarcoplasmic reticulum) may link telethonin to calcium metabolism [16], and its interaction with minK, a potassium channel β subunit, may also affect electromechanical coupling (or vice versa [17–20]). In addition, telethonin was shown to interact with MDM2 [21] and MuRF1 [22]—E3 ubiquitin ligases important for cardiac protein turnover as well as with the pro-apoptotic protein Siva [23]. Particularly, telethonin’s interaction with MDM2, which is important for p53 degradation—an important inductor of apoptosis—and with Siva, may point to a role of telethonin in the regulation of cell survival pathways.

Over the last decade, many different telethonin mutations have been described, including recessive nonsense mutations which are associated with limb-girdle muscular dystrophy type 2 (LGMD2G) [24–26] and heterozygous missense mutations which are associated with dilated (DCM) and hypertrophic (HCM) forms of cardiomyopathy [7, 27, 28] as well as with intestinal pseudo-obstruction [29]. Interestingly, a naturally occurring telethonin variant that has a Glu13 deletion (E13del telethonin) was initially found in patients affected by HCM [27] and then later in healthy, unaffected individuals [30, 31], but the molecular consequences of this variant, which renders telethonin unable to interact with titin, have been studied only recently [32].

While nonsense mutations in the telethonin gene are clearly linked to LGMD2G, the missense mutations found in this gene were identified by candidate gene approaches and linkage data are missing. To gain more insight into the underlying molecular mechanisms, we analysed telethonin’s in vivo function in the myocardium by replacing telethonin exons 1 and 2 with a lac Z neomycin cassette. Importantly, homozygous deficient telethonin knockout animals are born in the expected Mendelian ratios and are fertile.

The analysis of myocardial function in these animals by echocardiography as well as by in vivo heart catheterization under spontaneous conditions did not reveal any abnormal parameters. Also, immunohistochemistry and immunogold electron microscopy did not show any alterations in telethonin-deficient Z-discs. However, stretch of myofibrils following actin removal in telethonin-deficient heart and skeletal muscles clearly showed the importance of telethonin for titin localization. We also increased biomechanical stress under in vivo conditions by transverse aortic constriction (TAC) and in another set of experiments we treated telethonin^−/−^ animals with doxorubicin, an inductor of oxygen radicals. After these interventions, telethonin^−/−^ developed maladaptive cardiac hypertrophy and severe heart failure owing at least in part to an increase in apoptosis.

Apoptosis can be efficiently induced by the tumour suppressor gene product p53, a protein known to be polyubiquitinylated and marked for degradation by the E3 ubiquitin ligase MDM2. Gene expression arrays identified increased levels of p53 in the telethonin^−/−^ myocardium following TAC and we also found significant increases in p21 and caspase 8 mRNAs, both of which are p53 target genes. Moreover, a significant increase in nuclear p53 was also observed.

Immunoprecipations, pull down and overlay assays together with static light scattering and NMR analysis confirmed that telethonin interacts with the p53 DNA-binding domain (p53DBD). Also, a series of immunofluorescence studies identified the colocalization of telethonin with p53 in cardiomyocyte nuclei under biomechanical as well as oxidative stresses.

We also aimed to analyse the effects of telethonin overexpression on myocardial function under in vivo conditions and generated telethonin transgenic animals. Here, we employed the myocardium-specific alpha myosin heavy chain promoter and a FLAG-tagged mouse telethonin cDNA. Again, these animals did not exhibit any spontaneous phenotype. Of note, they develop less apoptosis as well as less p53 expression in comparison with wild-type littermate controls after TAC. This finding is particularly interesting and might indicate potential protective effects of telethonin overexpression and may lend support for the idea of using telethonin like peptides to influence cardiac plasticity following various types of biomechanical stresses.

We also studied telethonin mRNA expression in human hearts. Here, we analysed myocardial samples from end-stage heart failure patients and found significant telethonin downregulation in comparison with normal donor hearts together with an increase in nuclear telethonin. This finding may have implications for p53 expression and p53-related apoptosis, both of which have previously been shown to be elevated in these hearts [33]. We also found downregulation of telethonin in acute donor organ failure, suggesting that this effect is not restricted to the setting of chronic end-stage failure.

Besides telethonin, many other Z-disc proteins such as Ankrd2 (see below), MLP or FHL2 shuttle into the nucleus and probably function as co-factors of transcription [34–36]. In addition, the sarcomeric Z-disc harbours a number of important transcription factors, such as nuclear factor of activated T-cells (NFAT), which has been implicated in the onset of maladaptive myocardial hypertrophy.

Telethonin is not the only Z-disc/sarcomeric protein able to interact with p53—for example, Ankrd1/CARP [37] and Ankrd2 [20] can do so as well. Ankrd1 belongs to the family of muscle ankyrin repeat proteins (MARP) is localized to the I-band of the sarcomere and has been implicated in titin related stretch sensing [38]. Upon stretch, Ankrd1 translocates to the nucleus, where it interacts with and increases the activity of p53 [37]. Ankrd1 mutations have been discovered in HCM [39] and in DCM patients [40, 41], but the underlying molecular mechanisms remain not well understood. However, it is likely that its interaction with p53 and a possible role in the regulation of apoptosis may well play a role.

Ankrd2, which is related to Ankrd1, interacts with telethonin and, like Ankrd1, also has been shown to function as a link between the sarcomere and the nucleus, where it is able to interact with and to increase p53 activity.

The importance of p53 for myocardial adaptation to various forms of biomechanical forms of stress has been demonstrated recently in several models. For example, p53 is anti-angiogenic by inhibiting hypoxia inducible factor 1 and therefore involved in the transition from hypertrophy to heart failure [42]. Also during ischaemia/reperfusion p53 inhibits mitophagy, an anti-apoptotic process, which finally leads to apoptosis and heart failure [43]. p53 also has been shown to have detrimental effects in diabetic cardiomyopathy, where p53 leads to the production of reactive oxygen species (ROS) and contributes to cardiac dysfunction [44].

## Apoptosis, Sarcomeroptosis and Mechanoptosis

Stretch-induced apoptosis has been observed in various models, both under in vitro and in vivo conditions. For example, stretch of smooth muscle cells led particularly to the activation of the integrin β1—rac—p38 MAPK and p53 pathway [45, 46]. Also, apoptosis following various forms of biomechanical stress in form of ischaemia/reperfusion or TAC has been described many times since its initial description in the heart [47] (please see for a recent review [48])—however, the molecular events, which link biomechanical stress ultimately to the execution of apoptosis as a consequence of these conditions, remains unclear (Table 1).

**Table 1:** Summarizes some of the differences between apoptosis, sarcomeroptosis and mechanoptosis

	Apoptosis	Sarcomeroptosis	Mechanoptosis
Cell types	Every cell type	Actively contracting cells (i.e. mainly striated muscle cells)	Actively contracting cells (i.e. mainly striated muscle cells)
Dependent on	Various	Sarcomeres	Z-disc (at least sarcomere) dependent
Characterized by	Various	Interference of striated muscle cell proteins with mediators of apoptosis	Various
Trigger	Various	Various	Two different triggers:
			1) External: mechanical stress, hemodynamic overload
			2) Internal: actively contracting myocytes (loss of sarcomere activity and increased sarcomere activity)

While ischaemia/reperfusion and TAC have many features in common, such as ischaemia and hemodynamic overload, they are also distinct—for example: conditions of ischaemia/reperfusion can not only be induced in muscle cells but the hemodynamic overload imposed on the myocardium via TAC is specific for striated muscle cells. Apoptosis is a general suicide programme which can be observed in almost every cell type. However, cardiac and skeletal muscle cells can specifically interfere, for example, via telethonin which affects significantly p53 function and activity. It is an intriguing possibility that telethonin, due its position at the Z-disc, translates the increase in sarcomere activity, which can be observed during TAC, into pro-survival signals. This will also indicate that forces generated by the sarcomere are translated into survival pathways and that this form of anti-apoptotic signalling can only be found in actively contracting cells, such as cardiac and skeletal muscles. One can describe this type of apoptosis as sarcomeroptosis because the sarcomere, in form of telethonin, is able to specifically interefere with apoptosis. It may also make sense to differentiate a second type of apoptosis which is induced via mechanical stress and which may be called mechanoptosis [32].

By definition, sarcomeroptosis can only happen if sarcomeres are present, hence during early stages of embryonic development this type of cell death cannot be observed. In this context, it is probably also interesting to note that telethonin deficiency, under spontaneous conditions, is not associated with any obvious phenotype—muscle cells have to be challenged in order to unmask the sarcomeroptosis or mechanoptosis phenotype. Therefore, one possible conclusion is that sarcomeroptosis plays only a role after birth when sarcomeres are well developed, begin to contract and when the heart is able to sustain circulation on its own and during stress conditions.

It might well be important to differentiate sarcomeroptosis, which only occurs in striated muscles, from other forms of apoptosis which may occur in any other cell type. It also may be possible to differentiate external mechanoptosis, which can be found in various cell types after mechanical stimulation, from intrinsic types of mechanoptosis, which may be caused by changes in sarcomeric force production and which is a special type of sarcomeroptosis (Table 1).

It remains to be elucidated whether mutations in Z-disc proteins, which give rise to Z-discopathies [1], can be differentiated via their effects on pro-survival and pro-apoptotic pathways from mutations in sarcomeric proteins, which may give rise to sarcomeropathies. It also remains to be elucidated to which extent sarcomeroptosis and mechanoptosis are different from various other types of cell death such as macro-autophagy and autophagy [49], entosis [50] or necroptosis [51]. It might well be that tumour necrosis factor alpha receptor (TNFαR) signalling, which can differentiate between NFκB and pro-survival signalling, apoptosis and necroptosis [52], also plays a role here. Another important point is that p53 involves the transcriptional machinery to execute programmes of cell death, whereas activation of TNFαR activates a signal-transduction cascade directly leading to cell death—these differences may be useful to distinguish various types of cell death even further. Also, it is an intriguing possibility to use telethonin-like peptides to modify sarcomeroptosis and mechanoptosis and possibly develop novel paradigms for therapy.

## Summary

Telethonin-deficient animals, although displaying a primary defect in an integral Z-disc component, are not associated with any severe spontaneous cardiac or skeletal muscle phenotype.

However, biomechanical stress in the form of TAC, which causes an increase in afterload, or doxorubicin treatment, which induces oxygen radicals, cause a maladaptive response ultimately leading to global heart failure. At the molecular level, telethonin leads to an increase of p53 which is likely to cause the elevated apoptosis observed following various forms of biomechanical stress in these animals. Telethonin binds to p53 and is involved in MDM2-mediated degradation of this protein and therefore may serve as a pivotal element in cardiac signalling by controlling apoptosis and cell death via p53.

In addition, by translocating into the nucleus and by binding to p53’s DNA-binding domain, telethonin is potentially able to interfere with gene regulation and to repress the function of this important transcription factor (i.e. to act as a co-factor of transcription).

Therefore, telethonin, which is a muscle-specific protein and which is localized to the sarcomeric Z-disc, may well be involved in the translation of sarcomere activity into pro-survival pathways. The interference of muscle-specific proteins with mediators of apoptotic cell death may help to define novel types of cell death such as sarcomeroptosis and mechanoptosis.

The sarcomeric Z-disc, recently described as a “hot spot” for cardiomyopathy causing mutations [53] and by linking myofilament activity, mechanosensation, mechanotransduction and transcriptional activity (Z-disc transcriptional coupling), may well act as a central structure for cardiac adaptation to biomechanical stress [1]. This aspect may also help to understand the evolution of various diseases, including Z-discopathies and cardiomyopathies.

## Abbreviations

DCM: Dilated cardiomyopathy
HCM: Hypertrophic cardiomyopathy
ILK: Integrin linked kinase
LGMD2G: Limb-girdle muscular dystrophy type 2
MLP, CSRP3: Muscle LIM protein
NFAT: Nuclear factor of activated T-cells
TAC: Transverse aortic constriction
TNFαR: Tumour necrosis factor α receptor
